# Combination curcumin and (−)-epigallocatechin-3-gallate inhibits colorectal carcinoma microenvironment-induced angiogenesis by JAK/STAT3/IL-8 pathway

**DOI:** 10.1038/oncsis.2017.84

**Published:** 2017-10-02

**Authors:** G Jin, Y Yang, K Liu, J Zhao, X Chen, H Liu, R Bai, X Li, Y Jiang, X Zhang, J Lu, Z Dong

**Affiliations:** 1 Department of Pathophysiology, School of Basic Medical Sciences, Zhengzhou University, Zhengzhou, Henan, China; 2 Collaborative Innovation Center of Henan Province for Cancer Chemoprevention, Zhengzhou, Henan, China; 3 Laboratory of Bone Tumor, Henan Luoyang Orthopedic Hospital, Zhengzhou, Henan, China; 4 Department of Pathology, Henan Cancer Hospital, Zhengzhou University, Zhengzhou, Henan, China

## Abstract

Tumor microenvironment has a crucial role in cancer development and progression, whereas the mechanism of how it regulates angiogenesis is unclear. In this study, we simulated the colorectal carcinoma microenvironment by conditioned medium (CM) of colorectal carcinoma cell lines (SW620, HT-29, HCT116) supernatant or colorectal carcinoma tissue homogenate supernatant to induce normal endothelial cells (NECs). We found that colorectal carcinoma CM promoted tumor angiogenesis by coercing NECs toward tumor endothelial cells (TECs) with the activation of the JAK/STAT3 signaling pathway. Antibody array analysis showed HT-29 supernatant contained numerous angiogenesis-related proteins, especially IL-8. Interestingly, the production of IL-8 in NECs induced by HT-29 CM was also increased. We also verified the crucial role of IL-8 in promoting the CM-induced angiogenesis, as IL-8 repression by neutralizing antibody abolished the transition of NECs toward TECs. Curcumin and (−)-epigallocatechin-3-gallate (EGCG) are broadly investigated in cancer chemoprevention. However, poor bioavailability hurdles their application alone, and the mechanism of their anti-angiogenesis still need to be illuminated. Here, we found that curcumin combination with EGCG attenuated the tumor CM-induced transition of NECs toward TECs by inhibiting JAK/STAT3 signaling pathway. Furthermore, the combination of curcumin and EGCG markedly reduced tumor growth and angiogenesis in the colorectal carcinoma PDX mouse model, and the combined anti-angiogenic effect was better than that of curcumin or EGCG alone. Taken together, our findings provide a new mechanism of tumor angiogenesis, and the combination of curcumin and EGCG represents a potential anti-angiogenic therapeutic method for colorectal carcinoma.

## Introduction

Tumor microenvironment is being increasingly recognized as a key factor in cancer aggressiveness.^1^ However, the mechanism of how tumor microenvironment governs tumor vessel formation is still controversial. One widely accepted concept for neo-vessel growth in malignancy is that the tumor microenvironment-induced new vessels sprout from the existing vasculature.^2^ Nonetheless, compared with their normal counterparts, tumor vessels are aberrant in almost all aspects of their structure and function. The tumor vessels are heterogeneous and tortuous, branch chaotically and have uneven vessel lumens.^3^ Also, a lot of evidence indicated that there were many differences at the molecular and functional levels between tumor endothelial cells (TECs) and normal endothelial cells (NECs).^4, 5^ Take into account these studies, we wondered whether the tumor microenvironment promoted angiogenesis by coercing NECs change toward TECs, and resulted in the heterogeneity of TECs.

Colorectal carcinoma is the third most common cancer in the world, and current therapeutics have only modest efficacy as the high metastasis and recurrence rate.^6^ Recent study demonstrated that p-STAT3 overexpression was detected in patients with colorectal carcinoma, and was associated with the metastasis and poor prognosis in colorectal carcinoma patients.^7^ In addition, The JAK/STAT3 (Janus kinase/signal transducer and activator of transcription 3) pathway is an important oncogenic signaling cascade that regulates many important biological functions, including cell proliferation, differentiation, immune response and angiogenesis.^8, 9^ However, whether the JAK/STAT3 signaling pathway was activated during the formation of TECs in colorectal carcinoma has not been researched.

Curcumin is a natural polyphenol from turmeric, and (−)-epigallocatechin-3-gallate (EGCG) is the most abundant and active component of green tea.^10, 11^ Previous researches have indicated that both curcumin and EGCG had chemopreventive effect on inhibiting the initiation and development of cancer.^12^ Accumulating research indicated that EGCG exerted anti-cancer property by suppressing the STAT3 signaling pathway.^13^ Curcumin was supposed to be a potent inhibitor of STAT3, which is a transcription factor, which has a role in tumorigenesis of many human malignancies.^14^ However, the poor absorption and low bioavailability of curcumin restrict its clinical application.^15^ Notably, recent studies demonstrated that curcumin and EGCG had synergistic activity with other drugs.^12, 16^ The approach of combination therapy has been shown to achieve higher therapeutic efficacy with lower drug dosage and reduce drug resistance development.^17^ Therefore, we speculated that combination of naturally derived agents curcumin and EGCG might produce a better anti-colorectal carcinoma effect.

Based on the above consideration, we simulated the colorectal carcinoma microenvironment by colorectal carcinoma cell lines (HT-29, SW620 and HCT116) or tissue homogenate supernatant that were temporarily called tumor conditioned medium (CM) to investigate the influence on NECs, and further explored the mechanism of tumor angiogenesis. Then, we investigated the combined inhibitory effect of curcumin and EGCG on the CM-induced transition of NECs toward TECs. Finally, we detected the combined anti-angiogenic effect of curcumin and EGCG *in vivo* by colorectal carcinoma patient-derived xenograft (PDX) mouse model.

## Results

### Colorectal carcinoma CM promotes the migration, invasion, tube formation and Dil-Ac-LDL uptake abilities of NECs

**Figure 1:**
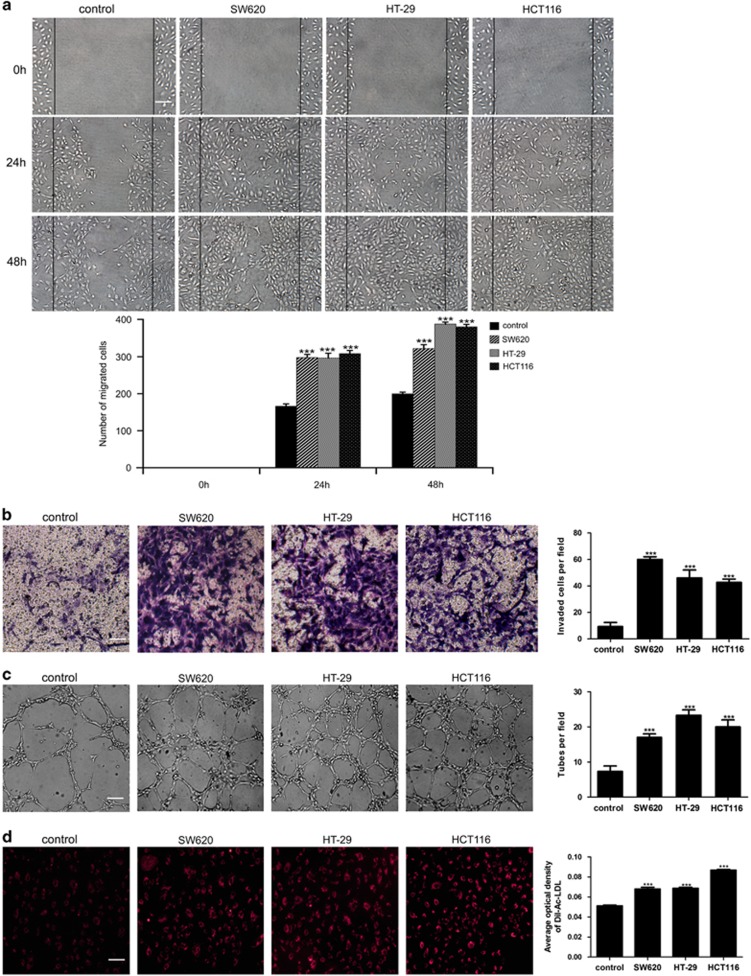
Colorectal carcinoma CM enhanced the migration, invasion, tube formation and Dil-Ac-LDL uptake abilities of NECs. (**a**) NECs monolayer was wounded and induced by SW620, HT-29 or HCT116 CM. Photographs were taken after induction for 0, 24 and 48 h (scale bar 40 μm). (**b**) NECs were induced by SW620, HT-29 or HCT116 CM for 48 h. The number of invaded cells was counted in three random fields. Representative images of invaded cells were shown (scale bar 40 μm). (**c**) Formation tubes in each group were photographed. The number of tubes per field was counted in three random fields (scale bar 20 μm). (**d**) Representative images showed the Dil-Ac-LDL uptake ability of NECs and quantification of the relative Dil-Ac-LDL uptake (scale bar 40 μm). Data are presented as mean±s.d. from three independent experiments. ***P<0.001.

As migration and invasion of endothelial cells (ECs) are two key steps for the formation of new blood vessels, we performed wound-healing and transwell assays to determine the effects of colorectal carcinoma CM on the migration and invasion abilities of NECs. The results showed that SW620, HT-29 or HCT116 CM promoted the migration and invasion of NECs (Figures 1a). To further investigate the effect of tumor CM on angiogenesis, we performed tube formation assay. We found that SW620, HT-29 or HCT116 CM markedly promoted the tube formation ability of NECs (Figure 1c). Interestingly, the Dil-labeled acetylated low-density lipoprotein (Dil-Ac-LDL) uptake ability of NECs was also increased after induction by CM of SW620, HT-29 or HCT116 (Figure 1d). Human colorectal carcinoma tissue homogenate CM was further used to simulate the tumor microenvironment. The results showed that the migration, invasion, tube formation and Dil-Ac-LDL uptake abilities of NECs were remarkably enhanced after induction by colorectal carcinoma tissue homogenate CM, compared with that of induced by peri-carcinoma tissue homogenate CM (Supplementary Figure 1). These data suggest that colorectal carcinoma microenvironment promotes the initiation of angiogenesis by enhancing the angiogenic properties of NECs.

### Colorectal carcinoma CM promotes the transition of NECs toward TECs

**Figure 2:**
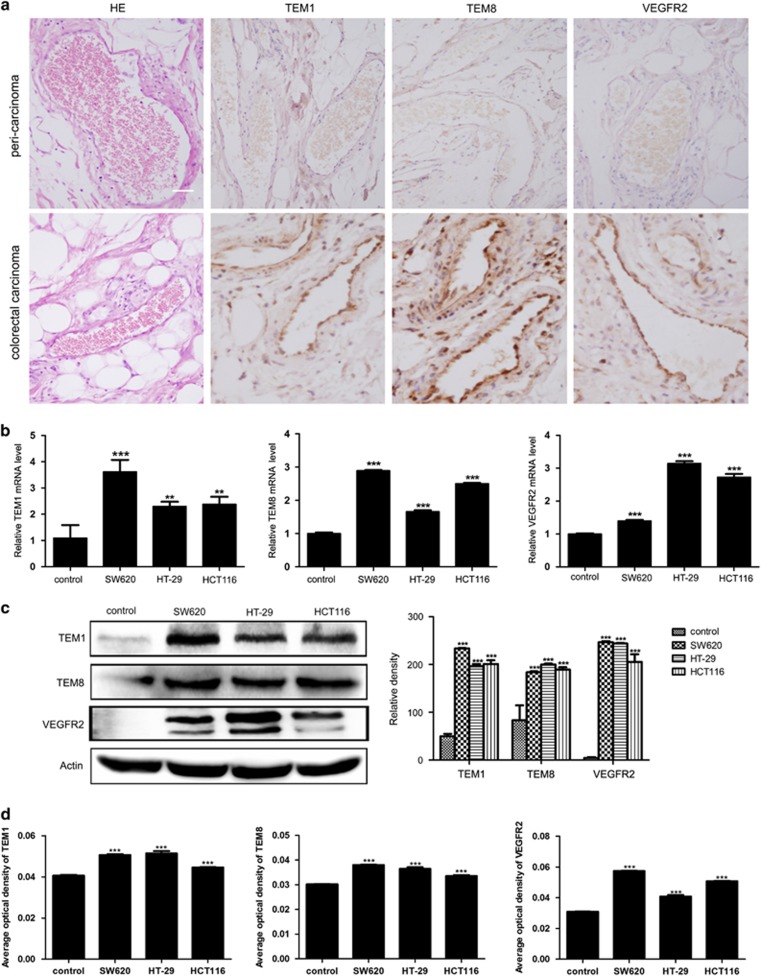
Colorectal carcinoma CM promoted the transition of NECs toward TECs. (**a**) Immunohistochemical staining for TECs markers (TEM1, TEM8 and VEGFR2) in colorectal carcinoma and peri-carcinoma tissue (scale bar 20 μm). (**b**) NECs were induced by SW620, HT-29 or HCT116 CM for 48 h. The relative mRNA levels of TECs markers were determined by qRT–PCR. (**c**, **d**) NECs were induced by SW620, HT-29 or HCT116 CM for 48 h, the protein levels of TECs markers were detected by western blot (**c**) and immunofluorescence (**d**). Data are presented as mean±s.d. from three independent experiments. **P<0.01, ***P<0.001.

It has been proved that TECs possessed a distinct phenotype, and different from NECs at both molecular and functional levels.^18^ Based on the above observation that colorectal carcinoma CM enhanced the angiogenic properties of NECs, we wondered whether colorectal carcinoma CM promoted angiogenesis by switching NECs toward TECs. To test this hypothesis, we first performed immunohistochemistry assay on paraffin section of human colorectal carcinoma and peri-carcinoma tissue from patients to detect the expression of selected TECs markers. The results showed that TEM1, TEM8 and VEGFR2 were specifically expressed in vascular ECs of carcinoma tissue (Figure 2a). These results further confirmed that TEM1, TEM8 and VEGFR2 were TECs markers, which were consistent with previous reports.^19, 20^ Then, we detected the expression levels of TECs markers in CM-induced NECs. We found that SW620, HT-29 or HCT116 CM-induced NECs expressed TECs markers higher both in mRNA and protein levels compared with NECs in the control group (Figures 2b–d). For NECs induced by colorectal carcinoma tissue homogenate CM, the results were in accordance with that induced by colorectal carcinoma cell lines CM. NECs in colorectal carcinoma tissue homogenate CM-induced group expressed TECs markers higher than NECs in peri-carcinoma tissue homogenate CM-induced group (Supplementary Figure 2). These results suggest that colorectal carcinoma microenvironment promotes the transition of NECs toward TECs.

### JAK/STAT3 signaling pathway is activated during the transition of NECs toward TECs induced by colorectal carcinoma CM

**Figure 3:**
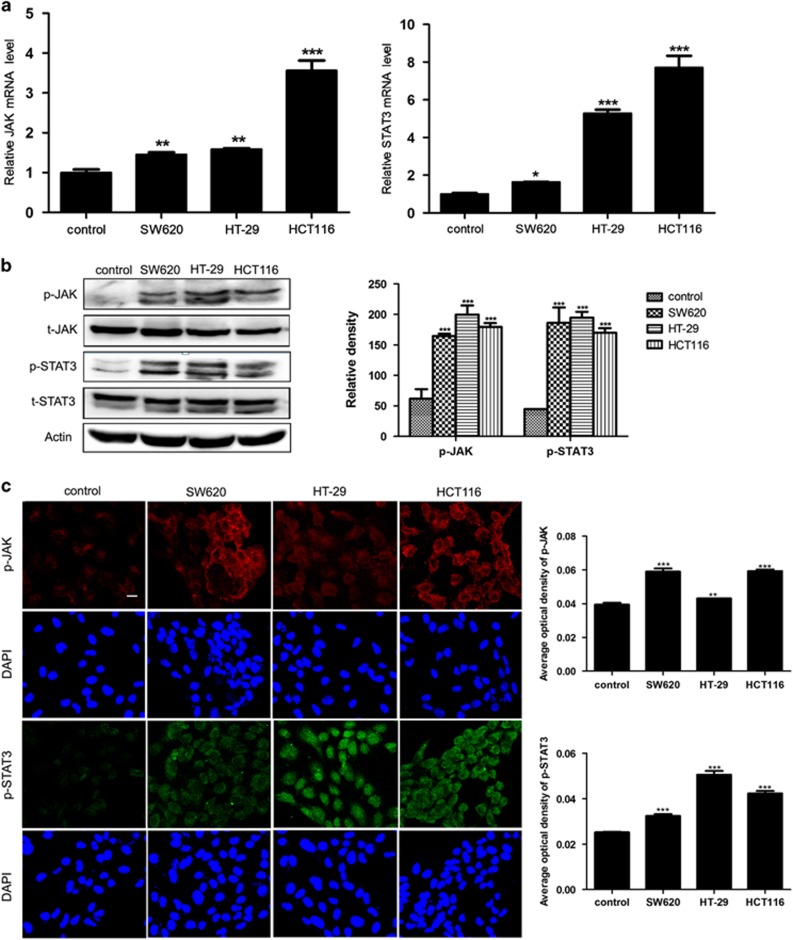
JAK/STAT3 signaling pathway was activated during the transition of NECs toward TECs induced by colorectal carcinoma CM. (**a**) NECs were induced by SW620, HT-29 or HCT116 CM for 48 h. The relative mRNA levels of JAK and STAT3 were measured by qRT–PCR. (**b**, **c**) The expression level of indicated protein was detected by western blot (**b**) and immunofluoresence (scale bar 50 μm) (**c**). Data are expressed as mean±s.d. from three independent experiments. *P<0.05, **P<0.01, ***P<0.001.

It has been noted that persistent activation of JAK/STAT3 signaling pathway was implicated in many aspects of tumorigenesis affected by tumor microenvironment.^21, 22^ However, whether JAK/STAT3 signaling pathway was activated during the transition of NECs toward TECs induced by colorectal carcinoma microenvironment was unclear. In this study, we found that after induction by SW620, HT-29 or HCT116 CM, the relative JAK and STAT3 mRNA levels in CM-induced NECs were significantly increased compared with control NECs (Figure 3a). Furthermore, the phosphorylated JAK and STAT3 protein levels also remarkably increased in CM-induced NECs of SW620, HT-29 and HCT116 group (Figures 3b and c). Colorectal carcinoma tissue homogenate CM was further used to induce NECs. The results were in accordance with that induced by colon cancer cell lines CM as expected (Supplementary Figure 3). These data insinuate that JAK/STAT3 is activated during the transition of NECs toward TECs induced by colorectal carcinoma CM.

### IL-8 in HT-29 CM has a key role in promoting the transition of NECs toward TECs

**Figure 4:**
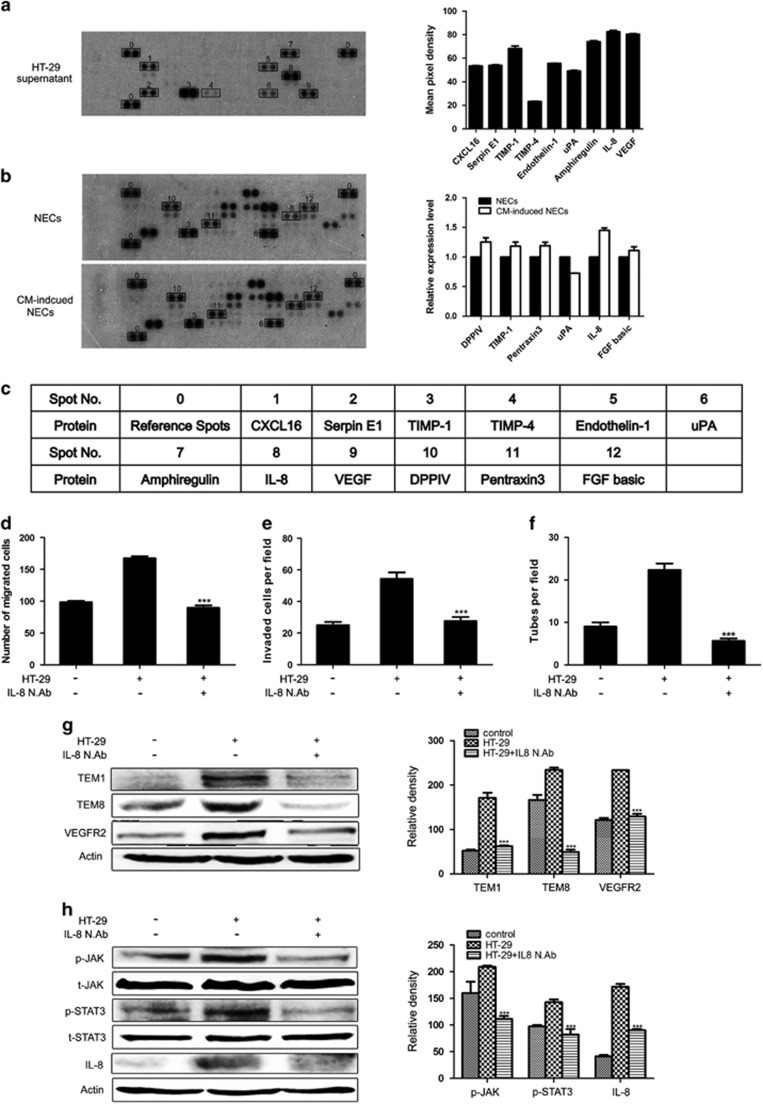
IL-8 had a key role during the transition of NECs toward TECs. (**a**) Proteome profiler array detected multiple human angiogenesis-related proteins in HT-29 supernatant. Nine angiogenesis-related proteins that expressed at relatively high level were highlighted with squares and measured the mean pixel density. (**b**) Proteome profiler array detected the angiogenesis-related protein levels in NECs and HT-29 CM-induced NECs. Differently expressed proteins in HT-29 CM-induced NECs compared with NECs were highlighted with squares and indicated by numbers. Each spot was spotted on the array membrane in duplicate. (**c**) The numbers in **a** and **b** represented the corresponding proteins. (**d**–**f**) NECs were induced by HT-29 CM pretreated with IL-8-neutralizing antibody (N. Ab) or not for 48 h. Then the migration, invasion and tube formation abilities of NECs were measured by wound-healing assay (**d**), transwell assay (**e**), tube formation assay (**f**). (**g**, **h**) NECs were induced by certain CM for 48 h. The expression levels of indicated proteins were detected by western blot. Data in **d**–**h** are presented as mean±s.d. from three independent experiments. ***P<0.001.

In an attempt to detect the key factor that promoted the transition of NECs toward TECs, a proteome profiler array was conducted on the HT-29 supernatant. Multiple human angiogenesis-related proteins were detected in HT-29 supernatant, such as VEGF, IL-8, Amphiregulin, TIMP-1, CXCL16 and so on (Figure 4a). To further investigate the effect of HT-29 CM on NECs, we analyzed angiogenesis-related proteins in NECs and HT-29 CM-induced NECs through a proteome profiler human angiogenesis antibody array. We found that HT-29 CM-induced NECs expressed IL-8 higher than control NECs (Figure 4b). The above results have revealed that HT-29 CM promoted the migration, invasion and tube formation of NECs. To confirm that this effect was indeed dependent on the preferential overexpression of IL-8 in HT-29 supernatant, an IL-8-neutralizing antibody was used to abolish the activity of IL-8. The results showed that the enhanced migration, invasion and tube formation abilities of HT-29 CM-induced NECs were eliminated by IL-8-neutralizing antibody (Figures 4d–f). The effect of IL-8 in HT-29 CM on the transition of NECs toward TECs was further investigated. The HT-29 CM-induced overexpression of TECs markers (TEM1, TEM8 and VEGFR2) in NECs were significantly blocked upon silencing IL-8 by neutralizing antibody (Figure 4g). Notably, IL-8 blocking abrogated the HT-29 CM-induced activation of JAK/STAT3 signaling pathway, and the production of IL-8 in NECs was also decreased (Figure 4h). Taken together, our data reveal that IL-8 secreted by tumor cells activates JAK/STAT3 signaling pathway and induces the production of IL-8 in NECs, and this amplification loop promotes the transition of NECs toward TECs.

### Combination of curcumin and EGCG inhibits the transition of NECs toward TECs by blocking JAK/STAT3/IL-8 signaling pathway

**Figure 5:**
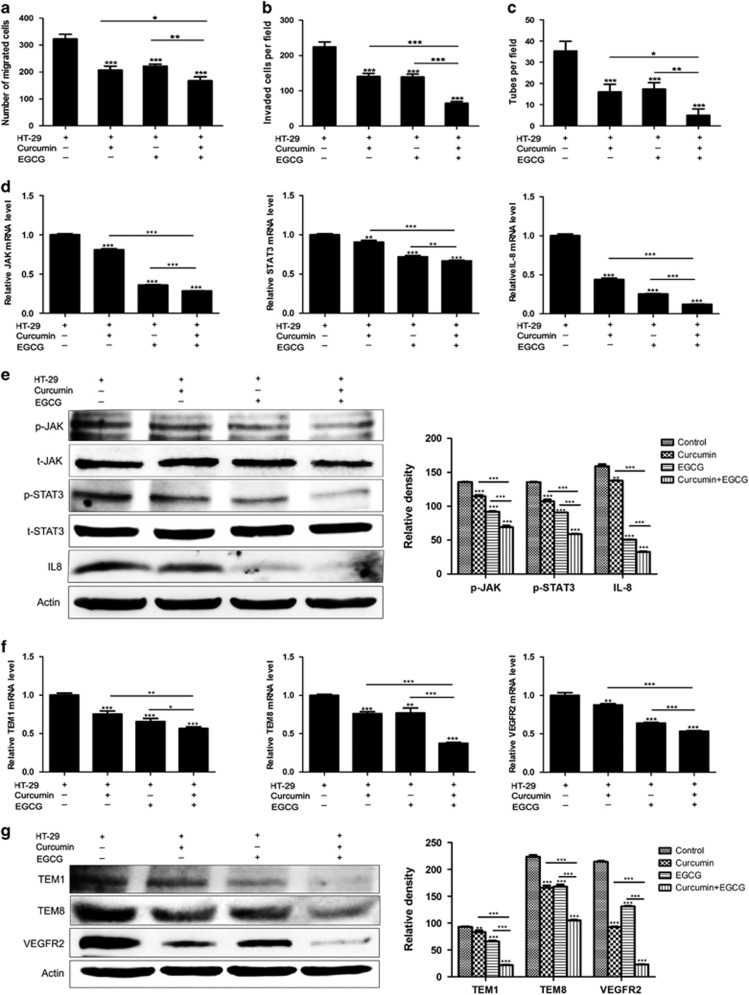
Combination curcumin and EGCG inhibited the HT-29 CM-induced transition of NECs toward TECs by blocking JAK/STAT3/IL-8 signaling pathway. (**a–c**) NECs were treated with curcumin, EGCG or curcumin combination with EGCG for 48 h during induction by HT-29 CM. The migration, invasion and tube formation abilities of HT-29 CM-induced NECs were examined by wound-healing assay (**a**), transwell assay (**b**) and tube formation assay (**c**). (**d**) The relative mRNA levels of JAK, STAT3 and IL-8 were examined by qRT–PCR. (**e**) Western blot was conducted for the indicated proteins. (**f**) The relative mRNA levels of TECs markers (TEM1, TEM8, VEGFR2) were examined by qRT–PCR. (**g**) The protein levels of TECs markers were detected by western blot. Results are expressed as mean±s.d. from three independent experiments. *P<0.05, **P<0.01, ***P<0.001.

Curcumin and EGCG are widely investigated chemopreventive candidates for cancer by inhibiting the JAK/STAT3 signaling pathway.^23, 24, 25^ However, the precise mechanism of their anti-angiogenic action and their combined effects has not been fully evaluated. In this research, NECs were treated with EGCG and curcumin alone or in combination during induced by HT-29 CM. We found that EGCG or curcumin inhibited the viability of NECs in a dose-dependent manner. Based on this result, lower concentration of EGCG (5 μM) and curcumin (50 μM) were selected and applied to investigate the synergistic effect of these two compounds. The enhanced migration, invasion and tube formation abilities of HT-29 CM-induced NECs were significantly abrogated by curcumin and EGCG. More importantly, combination of curcumin and EGCG had a stronger inhibitory effect than using each drug alone (Figures 5a–c). We have demonstrated that JAK/STAT3 signaling pathway was activated during the transition of NECs toward TECs induced by colorectal carcinoma microenvironment. We further assessed the influence of curcumin and EGCG on the activation of JAK/STAT3 signaling pathway. The results showed that the mRNA levels of JAK, STAT3 and IL-8 were decreased in curcumin and EGCG-treated group compared with the control group (Figure 5d). In addition, curcumin and EGCG reduced the protein levels of p-JAK, p-STAT3 and IL-8 in HT-29 CM-induced NECs. It is noteworthy that the combined inhibitory effect was more remarkable (Figure 5e and Supplementary Figure 4). Then, we evaluated the relative expression levels of TECs markers in HT-29 CM-induced NECs to explore the mechanism of the anti-angiogenic effect of curcumin and EGCG. The results showed that curcumin combination with EGCG markedly reduced the expression TEM1, TEM8 and VEGFR2 of HT-29 CM-induced NECs both at mRNA and protein levels (Figures 5f and g and Supplementary Figure 5). Collectively, these results provide evidence that combination of curcumin and EGCG abrogates HT-29 CM-induced transition of NECs toward TECs by blocking JAK/STAT3/IL-8 signaling pathway.

### Combination of curcumin and EGCG inhibits tumor angiogenesis in colorectal carcinoma PDX mouse model

**Figure 6:**
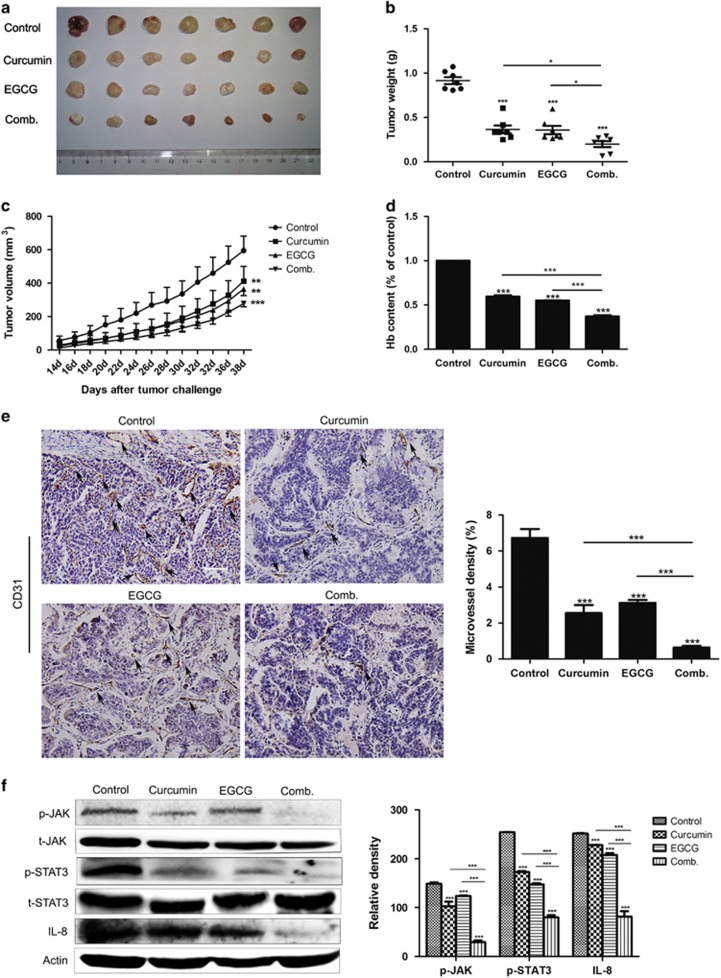
Combination of curcumin and EGCG suppressed tumor angiogenesis in colorectal carcinoma PDX mouse model. (**a**, **b**) PDX tumor-bearing mice were given curcumin, EGCG or curcumin combination with EGCG (Comb.) every other day for 4 weeks. The tumors were excised at the end of the experiment. The size and weight of tumors were measured. (**c**) The tumor volume was measured and recorded every other day. (**d**) The tumors excised from mice were quantified the hemoglobin content. (**e**) The tumors excised from mice were stained with CD31 and quantified microvessel density (scale bar 20 μm). (**f**) The levels of indicated protein in tumor specimens were assessed by western blot. Data in (**d**–**f**) are shown as mean±s.d. three independent experiments. *P<0.05, **P<0.01, ***P<0.001.

To further investigate the combined anti-angiogenic effect of curcumin and EGCG *in vivo*, we used human colorectal carcinoma PDX mouse model. Results showed that curcumin and EGCG suppressed the colorectal carcinoma PDX tumor growth. Notably, the anti-tumor effect was remarkably enhanced when treated mice with curcumin combination with EGCG (Figures 6a–c). Then the hemoglobin assay and immunohistochemical analysis were performed to confirm the anti-angiogenic effect of curcumin and EGCG. The results showed that the hemoglobin content and the microvessel density of tumor specimens in curcumin or EGCG group were decreased compared with the control group. More importantly, when treating mice with curcumin combined with EGCG, the hemoglobin content and the microvessel density in tumor specimens were reduced more remarkably than using signal compound (Figures 6d and e). Then, we measured the change of JAK/STAT3/IL-8 signaling pathway in the tumor specimens of mice. We observed that the protein levels of p-JAK, p-STAT3 and IL-8 in tumor specimens of curcumin combination with EGCG-treated group were decreased significantly, which corroborated our *in vitro* results (Figure 6f). Overall, these results further suggest that the combination of curcumin and EGCG inhibits colorectal carcinoma angiogenesis *in vivo* by blocking JAK/STAT3/IL-8-signaling pathway.

## Discussion

Angiogenesis, the process of new blood vessel growth, is necessary for tumor progression and metastasis.^26^ However, the mechanism of tumor angiogenesis has not yet been fully evaluated. Considering the growing awareness of that tumor microenvironment had an important role in tumor progression, we wondered whether the tumor microenvironment promoted angiogenesis by changing the phenotype and function of NECs. In this study, colorectal carcinoma microenvironment was simulated by colorectal carcinoma CM to induce NECs. We found that colorectal carcinoma CM enhanced the pro-angiogenic property of NECs, such as migration, invasion, tube formation and so on. Further study indicated that colorectal carcinoma CM-induced NECs expressed TECs markers at higher levels compared with NECs. These data provide evidence that colorectal carcinoma CM promoted tumor angiogenesis by transition NECs toward TECs. As it is hard to isolate TECs from tumor tissue, most studies related tumor angiogenesis were carried out by using NECs such as human umbilical vein endothelial cells or human dermal microvascular endothelial cells.^27, 28^ Our finding that NECs induced by tumor CM have the characteristic of TECs provides a novel method to conduct future research on tumor angiogenesis especially on drugs target to TECs.

To detect the key factors that promoted the change of NECs, we conducted the proteome profiler array on HT-29 supernatant. Among the detected angiogenesis-related proteins in HT-29 supernatant, IL-8 had a tendency to attract more attention. IL-8 is a proinflammatory cytokine that functions as a chemoattractant and is also a potent pro-angiogenic factor.^29^ It has been reported that the IL-8 level in the serum of cancer patients was highly elevated during tumor progression and contributed to the development of distant metastasis.^30, 31^ Given the above data that HT-29 CM promoted the transition of NECs toward TECs, we hypothesized that IL-8 in the tumor microenvironment might have a prominent role in this process. This hypothesis was verified by the subsequent experiments that the HT-29 CM-induced transition of NECs toward TECs was abolished when silencing IL-8 in HT-29 CM by IL-8-neutralizing antibody.

Previous studies have suggested that IL-8 promoted malignant tumor growth and metastasis in an autocrine and paracrine manner.^32, 33^ In addition, tumor-derived IL-8 activated ECs in the tumor vasculature to promote angiogenesis.^34^ Our subsequent proteome profiler array on the lysate of cells indicated that the HT-29 CM-induced NECs highly expressed IL-8 compared with NECs. Moreover, silencing IL-8 in HT-29 CM by IL-8-neutralizing antibody suppressed the JAK/STAT3 signaling pathway and decreased the protein level of IL-8 in HT-29 CM-induced NECs. It has been known that the secreted IL-8 from tumor cells binding to two cell-surface G protein-coupled receptors, termed CXCR1 and CXCR2, promoted activation of JAK/STAT3 signaling pathway and nuclear translocation of STAT3.^35^ Our data provided the evidence that IL-8 in the tumor microenvironment activated the positive feedback loop through JAK/STAT3 signaling and increased the expression of IL-8 in HT-29 CM-induced NECs. Therefore, we propose that inhibition of JAK/STAT3/IL-8 signaling pathway could be a significant therapeutic method against the transition of NECs toward TECs.

Curcumin and EGCG have been reported acted as multi-targeting agents in regulating JAK/STAT3 signaling in cancer.^23^ In addition, curcumin and EGCG have been used alone to demonstrated the anti-angiogenic effect.^36, 37^ However, the exact mechanism of their anti-angiogenic effect is still under controversy. Considering the poor absorption and low bioavailability of curcumin, and the synthesis anti-tumor effect of EGCG, the combination of curcumin and EGCG may produce a better anti-tumor effect.^38, 39^ In this research, we found that curcumin and EGCG significantly inhibited the migration, invasion and tube formation abilities of HT-29 CM-induced NECs. In addition, curcumin and EGCG inhibited HT-29 CM-induced transition of NECs toward TECs by suppressing the activation of JAK/STAT3/IL-8 signaling pathway. More importantly, the combined inhibitory effect was stronger than either single agent. Then, the colorectal carcinoma PDX mouse model was further used to explore the synergistic anti-angiogenic effect of curcumin and EGCG *in vivo*. It has been widely known that the PDX mouse model was highly relevant to real human tumor growth, and the tumors maintained the original molecular characteristic and heterogeneity.^40^ Thus, our results are more credible and have stronger predictive power for translating this knowledge from the bench to bedside. In colorectal carcinoma PDX model, curcumin and EGCG significantly suppressed the tumor growth and angiogenesis. Notably, the combined inhibitory effect is more remarkable. Considering the synergistic enhanced anti-angiogenic effect of curcumin and EGCG, the combination of curcumin and EGCG may provide a safe and effective strategy for treating colorectal carcinoma.

On the whole, we demonstrate for the first time that the colorectal carcinoma microenvironment promotes angiogenesis through coercing NECs toward TECs with the activation of JAK/STAT3/IL-8 signaling pathway. Curcumin and EGCG exert anti-angiogenic effect by blocking this process, and the combination of curcumin and EGCG is promised to be a beneficial anti-angiogenic therapeutic method.

## Materials and methods

### Cell culture

The human colorectal cancer cell lines SW620, HT-29 and HCT116 were obtained from Nanjing Keygen Biotech Corp. (Nanjing, China), and cultured in RPMI-1640 (Biological Industries, Kibbutz Beit Haemek, Israel) medium with 10% fetal bovine serum. Human umbilical vein endothelial cells were isolated from aseptic cords as previous report.^41^ This research was approved by the Ethics Committee of Zhengzhou University and the patients whose tumor sample involved in the study were completely informed and provided consent. Human umbilical vein endothelial cells were cultured in EC medium (ScienCell, Carlsbad, CA, USA) and used as control in this research. All the cell lines were incubated in an atmosphere of 5% CO_2_ at 37 °C, and tested regularly for mycoplasma contamination in our laboratory.

### Preparation of tumor CM

HT-29, SW620 or HCT116 were inoculated in 10-cm dishes, replenished with 5 ml fresh medium after reaching 60–80% confluence. After culturing for 24 h, the supernatant was collected and centrifuged at 3000 r.p.m., then stored at −20 °C. Cancer cell line CM was composed by 60% HT-29, SW620 or HCT116 supernatant and 40% fetal bovine serum-free EC medium. The colorectal carcinoma tissue homogenate supernatant was prepared as previously described.^42^ In brief, fresh tumor specimens of colorectal carcinoma tissue and the corresponding peri-carcinoma tissues were grinded and centrifuged at 13000 r.p.m. for 30 min, the supernatant was collected and stored at −20 °C. Colorectal carcinoma tissue homogenate CM was comprised by 40% supernatant and 60% fetal bovine serum-free EC medium. Patients whose tumor sample involved in this study were completely informed and provided consent.

### Wound-healing assay

NECs were planted in 12-well culture plates and incubated to a density of 60–70%. Then the cell monolayer was scratched with 200-μl pipette tip and washed twice with PBS. Colorectal carcinoma CM was applied to culture NECs for 48 h. The wounded areas were imaged by microscope (Olympus, Tokyo, Japan) at 0 h, 24 h and 48 h. The number of migrated cells in per field was counted.

### Transwell assay

Transwell migration chambers (Corning, NY, USA) were precoated with diluted matrigel (1:4, BD Biosciences, San Jose, CA, USA) and incubated at 37 °C for 2 h. Cells (3.75 × 10^4^ cells per well) were added to the upper chamber, and the lower chamber was filled with 750 μl completed EC medium. The chambers were incubated at 37 °C for 24 h. Then the invaded cells were counted after staining with crystal violet and imaged by microscope (Olympus).

### Tube formation assay

Chilled liquid matrigel was pipetted into 96-well plates (50 μl per well) and polymerized for 2 h at 37 °C. Cells (2 × 10^4^ cells per well) were seeded onto the gel and cultured at 37 °C for 4 h. The enclosed networks of complete tubes were photographed and counted in randomly selected fields.

### Dil-Ac-LDL uptake assay

NECs (3 × 10^3^ per well) were seeded in 96-well plates for adherent overnight. After induction by colorectal carcinoma CM for 48 h, Dil-Ac-LDL (10 μg/ml; Biomedical Technologies, Stoughton, MA, USA) was added to each well, and further incubated for 4 h. Then the cells were washed with PBS three times and photographed using IX2-IL100 fluorescence microscope (Olympus). The average optical density of Dil-Ac-LDL was calculated by Image J.

### Quantitative real-time PCR

Total RNA was extracted using TRIzol reagent (Invitrogen, Carlsbad, CA, USA). RNA concentration, integrity and quality were determined using NanoDrop 2000C (Thermo Fisher Scientific, Waltham, MA, USA). One microgram of total RNA was reverse transcribed into cDNA using a RT reagent kit (TaKaRa, Tokyo, Japan). Quantitative real-time PCR was carried out in triplicate with SYBR Premix Ex Taq (TaKaRa) using a 7500 Fast Real-time PCR System (Applied Biosystems, Grand Island, NY, USA) as recommended by the manufacturer. GAPDH was used as an internal control. The expression of individual gene was calculated and normalized with the 2^−ΔΔCt^ method.

### Western blot

Western blot was performed as previously described.^43^ In brief, total proteins were extracted from cells and separated using sodium dodecyl sulfate–polyacrylamide gel electrophoresis and then transferred to a polyvinyldifluoride membrane (Millipore, MA, USA). Then the membrane was incubated with a specific primary antibody at 4 °C overnight. The following antibodies were used in this study: anti-p-JAK2 (Tyr1007/Tyr 1008) (sc-16566-R, Santa Cruz, Dallas, TX, USA), anti-JAK2 (sc-294, Santa Cruz), anti-p-STAT3 (sc-8059, Santa Cruz), anti-STAT3 (sc-483, Santa Cruz), anti-TEM1 (sc-377221, Santa Cruz, anti-TEM8 (ab21270, Abcam, Cambridge, UK), anti-VEGFR2 (sc-321, Santa Cruz), anti-IL-8 (ab18672, Abcam) and anti-Actin (sc-8432, Santa Cruz). Protein bands were visualized using a chemiluminescence detection kit (Beyotime, Shanghai, China) after hybridization with a horseradish peroxidase-conjugated secondary antibody.

### Immunofluorescence

Immunofluorescence was performed as previously described.^44^ In brief, cells were fixed with 4% paraformaldehyde for 30 min at room temperature, permeabilized with 0.5% Triton X-100 for 10 min and blocked with 1% bovine serum albumin–phosphate-buffered saline with Tween 20 for 1.5 h. After that, cells were incubated with primary antibodies (1:50 diluted) overnight. The primary antibodies used in immunofluorescence were same with that used in Western bolt. Then the cells were washed with PBST and incubated with Rhodamine—Conjugated AffiniPure Goat Anti-Rabbit IgG (ZF-0316, Origene, Beijing, China) or Fluorescein-Conjugated AffiniPure Goat Anti-Mouse IgG (ZF-0312, Origene). All images were captured with a laser scanning confocal microscope (Olympus). The average optical density of individual protein was calculated with the formula: the value of Integrated density/the value of Area. The values of Integrated density and Area were measured by image J software.

### Human angiogenesis array kit/proteome profiler

Proteome Profle Human Antibody Array Kit (R&D Systems Ltd, Abingdon, UK) was used to analyze the expression profiles of angiogenesis-related proteins according to the manufacturer’s instruction. This array kit included 55 antibodies which directed to proteins involved in angiogenesis and invasiveness, and were spotted onto a nitrocellulose membrane in a duplicate way. The supernatant of HT-29 or protein extracted from NECs and HT-29 CM-induced NECs were mixed with 15 μl reconstituted detection antibody cocktail 1 h at room temperature. Then membranes were incubated with the sample/antibody mixtures overnight at 4 °C on a rocking platform. Following a washing step to remove unbound material, streptavidin-horseradish was added. Finally, membranes were exposed using enhanced chemiluminescence reagents and scanned into computer. The optical density of selected protein was measured by Image J software.

### Immunohistochemistry

The immunohistochemistry procedure was performed as described previously.^45^ The slides were incubated with primary antibody: anti-TEM1, anti-VEGFR2, anti-TEM8 or anti-CD31 (Abcam) at 4 °C overnight, then incubated HRP-IgG secondary antibody at 37 °C for 15 min. Coloring was performed with 3, 3′-diaminobenzidine and hematoxylin was used for counter staining. Hematoxylin–eosin staining was conducted according to standard histological procedures.

### Hemoglobin assay

Tumor tissue was weighted and homogenized in 1 ml RIPA lysis buffer. After centrifuging, 20 μl supernatant was added to 100 μl Drabkin’s solution (Sigma-Aldrich, St. Louis, MO, USA). The mixture was allowed to stand 30 min at room temperature, then absorbance at 540 nm was measured using an ELISA plate reader (Thermo Fisher Scientific, Waltham, CA, USA). The relative content of hemoglobin in the tumor tissue was compared with the control group.

### *In vivo* PDX study

Colorectal carcinoma PDX mouse model was established as reported previously.^46^ In brief, fresh human colorectal carcinoma fragments obtained intraoperatively from patients were subcutaneously implanted in female CB17/SCID mice of 6–8 weeks old. When the xenograft tumor reached ~1500 mm^3^, the mice were killed and tumors were divided into 0.1–0.2 g fragments and implanted into additional mice. After three consecutive mouse-to-mouse passages, tumor-bearing mice were randomly divided into four groups: Control, Curcumin, EGCG and Curcumin combination with EGCG (Comb.). No blinding was done for animal studies. Mice were given indicated drugs (50 mg/kg) every other day for 4 weeks. Mice were weighted and tumors were measured every other day. Tumor volume was calculated using the formula *V*=0.5*ab*^2^, with ‘*a*’ as the long diameter in millimeters and ‘*b*’ as the short diameter in millimeters. This research was approved by the Ethics Committee of Zhengzhou University and the patients whose tumor sample involved in the study were completely informed and provided consent. All experiments involved in mice were performed following the established guidelines of the Animal Ethics Committee of Zhengzhou University.

### Statistical analysis

Data were expressed as mean±s.d. Statistical significance for experiments with more than two subgroups was calculated by one-way ANOVA with Tukey’s *post hoc* test, when data were normally distributed. Comparisons between two subgroups were analyzed by *t*-test (two-sided).

For animal studies, sample size was estimated at seven mice per group to ensure power with statistical confidence. All statistical tests were performed using the Prism Software (GraphPad Software, San Diego, CA, USA). In all comparisons, *P*<0.05 was considered statistically significant.

## Publisher’s note:

Springer Nature remains neutral with regard to jurisdictional claims in published maps and institutional affiliations.

## References

[bib1] Zhou Z, Lu ZR. Molecular imaging of the tumor microenvironment. Adv Drug Deliv Rev 2016; 113: 24–48. 27497513 10.1016/j.addr.2016.07.012

[bib2] Weis SM, Cheresh DA. Tumor angiogenesis: molecular pathways and therapeutic targets. Nat Med 2011; 17: 1359–1370. 22064426 10.1038/nm.2537

[bib3] Magrini E, Villa A, Angiolini F, Doni A, Mazzarol G, Rudini N et al. Endothelial deficiency of L1 reduces tumor angiogenesis and promotes vessel normalization. J Clin Invest 2014; 124: 4335–4350. 25157817 10.1172/JCI70683 PMC4191010

[bib4] Hida K, Hida Y, Shindoh M. Understanding tumor endothelial cell abnormalities to develop ideal anti-angiogenic therapies. Cancer Sci 2008; 99: 459–466. 18167133 10.1111/j.1349-7006.2007.00704.x PMC11159852

[bib5] Hida K, Maishi N, Sakurai Y, Hida Y, Harashima H. Heterogeneity of tumor endothelial cells and drug delivery. Adv Drug Deliv Rev 2016; 99: 140–147. 26626622 10.1016/j.addr.2015.11.008

[bib6] Li X, Nie J, Mei Q, Han WD. MicroRNAs: novel immunotherapeutic targets in colorectal carcinoma. World J Gastroenterol 2016; 22: 5317–5331. 27340348 10.3748/wjg.v22.i23.5317 PMC4910653

[bib7] Ji K, Zhang MX, Chu Q, Gan Y, Ren H, Zhang LY et al. The role of p-STAT3 as a prognostic and clinicopathological marker in colorectal cancer: a systematic review and meta-analysis. PLoS ONE 2016; 11: e0160125. 27504822 10.1371/journal.pone.0160125 PMC4978497

[bib8] Braun DA, Fribourg M, Sealfon SC. Cytokine response is determined by duration of receptor and signal transducers and activators of transcription 3 (STAT3) activation. J Biol Chem 2013; 288: 2986–2993. 23166328 10.1074/jbc.M112.386573 PMC3561523

[bib9] Furtek SL, Backos DS, Matheson CJ, Reigan P. Strategies and approaches of targeting STAT3 for cancer treatment. Acs Chem Biol 2016; 11: 308–318. 26730496 10.1021/acschembio.5b00945

[bib10] Panahi Y, Darvishi B, Ghanei M, Jowzi N, Beiraghdar F, Varnamkhasti BS. Molecular mechanisms of curcumins suppressing effects on tumorigenesis, angiogenesis and metastasis, focusing on NF-kappa B pathway. Cytokine Growth Factor Rev 2016; 28: 21–29. 26774676 10.1016/j.cytogfr.2015.12.004

[bib11] Shin YS, Kang SU, Park JK, Kim YE, Kim YS, Baek SJ et al. Anti-cancer effect of (−)-epigallocatechin-3-gallate (EGCG) in head and neck cancer through repression of transactivation and enhanced degradation of beta-catenin. Phytomedicine 2016; 23: 1344–1355. 27765354 10.1016/j.phymed.2016.07.005

[bib12] Fajardo AM, Piazza GA. Chemoprevention in gastrointestinal physiology and disease. Anti-inflammatory approaches for colorectal cancer chemoprevention. Am J Physiol Gastrointest Liver Physiol 2015; 309: G59–G70. 26021807 10.1152/ajpgi.00101.2014 PMC4504955

[bib13] Tang SN, Fu J, Shankar S, Srivastava RK. EGCG enhances the therapeutic potential of gemcitabine and CP690550 by inhibiting STAT3 signaling pathway in human pancreatic cancer. PLoS ONE 2012; 7: e31067. 22348037 10.1371/journal.pone.0031067 PMC3278426

[bib14] Chung SS, Vadgama JV. Curcumin and epigallocatechin gallate inhibit the cancer stem cell phenotype via down-regulation of STAT3-NFkappaB signaling. Anticancer Res 2015; 35: 39–46. 25550533 PMC4290892

[bib15] Yin TF, Wang M, Qing Y, Lin YM, Wu D. Research progress on chemopreventive effects of phytochemicals on colorectal cancer and their mechanisms. World J Gastroenterol 2016; 22: 7058–7068. 27610016 10.3748/wjg.v22.i31.7058 PMC4988307

[bib16] Oya Y, Mondal A, Rawangkan A, Umsumarng S, Iida K, Watanabe T et al. Down-regulation of histone deacetylase 4, −5 and −6 as a mechanism of synergistic enhancement of apoptosis in human lung cancer cells treated with the combination of a synthetic retinoid, Am80 and green tea catechin. J Nutr Biochem 2017; 42: 7–16. 28103535 10.1016/j.jnutbio.2016.12.015

[bib17] Wagner H. Synergy research: approaching a new generation of phytopharmaceuticals. Fitoterapia 2011; 82: 34–37. 21075177 10.1016/j.fitote.2010.11.016

[bib18] Bussolati B, Grange C, Camussi G. Tumor exploits alternative strategies to achieve vascularization. FASEB J 2011; 25: 2874–2882. 21628445 10.1096/fj.10-180323

[bib19] St Croix B, Rago C, Velculescu V, Traverso G, Romans KE, Montgomery E et al. Genes expressed in human tumor endothelium. Science 2000; 289: 1197–1202. 10947988 10.1126/science.289.5482.1197

[bib20] Otsubo T, Hida Y, Ohga N, Sato H, Kai T, Matsuki Y et al. Identification of novel targets for antiangiogenic therapy by comparing the gene expressions of tumor and normal endothelial cells. Cancer Sci 2014; 105: 560–567. 24602018 10.1111/cas.12394 PMC4317838

[bib21] Bournazou E, Bromberg J. Targeting the tumor microenvironment: JAK-STAT3 signaling. JAKSTAT 2013; 2: e23828. 24058812 10.4161/jkst.23828 PMC3710325

[bib22] Kim BH, Yi EH, Ye SK. Signal transducer and activator of transcription 3 as a therapeutic target for cancer and the tumor microenvironment. Arch Pharm Res 2016; 39: 1085–1099. 27515050 10.1007/s12272-016-0795-8

[bib23] Casey SC, Amedei A, Aquilano K, Azmi AS, Benencia F, Bhakta D et al. Cancer prevention and therapy through the modulation of the tumor microenvironment. Semin Cancer Biol 2015; 35: S199–S223. 25865775 10.1016/j.semcancer.2015.02.007 PMC4930000

[bib24] Wu LC, Guo LX, Liang YH, Liu X, Jiang LH, Wang LS. Curcumin suppresses stem-like traits of lung cancer cells via inhibiting the JAK2/STAT3 signaling pathway. Oncol Rep 2015; 34: 3311–3317. 26397387 10.3892/or.2015.4279

[bib25] Wang ZW, Dabrosin C, Yin X, Fuster MM, Arreola A, Rathmell WK et al. Broad targeting of angiogenesis for cancer prevention and therapy. Semin Cancer Biol 2015; 35: S224–S243. 25600295 10.1016/j.semcancer.2015.01.001 PMC4737670

[bib26] Folkman J. Role of angiogenesis in tumor growth and metastasis. Semin Oncol 2002; 29: 15–18. 10.1053/sonc.2002.37263 12516034

[bib27] Michelini FM, Lombardi MG, Bueno CA, Berra A, Sales ME, Alche LE. Synthetic stigmasterol derivatives inhibit capillary tube formation, herpetic corneal neovascularization and tumor induced angiogenesis antiangiogenic stigmasterol derivatives. Steroids 2016; 115: 160–168. 27623061 10.1016/j.steroids.2016.09.001

[bib28] Wang JC, Li GY, Wang YC, Tang SC, Sun X, Feng XF et al. Suppression of tumor angiogenesis by metformin treatment via a mechanism linked to targeting of HER2/HIF-1 alpha/VEGF secretion axis. Oncotarget 2015; 6: 44579–44592. 26625311 10.18632/oncotarget.6373 PMC4792577

[bib29] Xie KP. Interleukin-8 and human cancer biology. Cytokine Growth Factor Rev 2001; 12: 375–391. 11544106 10.1016/s1359-6101(01)00016-8

[bib30] Benoy IH, Salgado R, Van Dam P, Geboers K, Van Marck E, Scharpe S et al. Increased serum interleukin-8 in patients with early and metastatic breast cancer correlates with early dissemination and survival. Clin Cancer Res 2004; 10: 7157–7162. 15534087 10.1158/1078-0432.CCR-04-0812

[bib31] Ren Y, Poon RTP, Tsui HT, Chen WH, Li Z, Lau CL et al. Interleukin-8 serum levels in patients with hepatocellular carcinoma: correlations with clinicopathological features and prognosis. Clin Cancer Res 2003; 9: 5996–6001. 14676125

[bib32] Guzman EA, Harmody D, Pitts TP, Vera-Diaz B, Winder PL, Yu Y et al. Inhibition of IL-8 secretion on BxPC-3 and MIA PaCa-2 cells and induction of cytotoxicity in pancreatic cancer cells with marine natural products. Anticancer Drugs 2016; 28: 153–160. 10.1097/CAD.0000000000000443 PMC9553068 27749658

[bib33] Tang KH, Ma S, Lee TK, Chan YP, Kwan PS, Tong CM et al. CD133(+) liver tumor-initiating cells promote tumor angiogenesis, growth, and self-renewal through neurotensin/interleukin-8/CXCL1 signaling. Hepatology 2012; 55: 807–820. 21994122 10.1002/hep.24739

[bib34] Waugh DJJ, Wilson C. The interleukin-8 pathway in cancer. Clin Cancer Res 2008; 14: 6735–6741. 18980965 10.1158/1078-0432.CCR-07-4843

[bib35] Holmes WE, Lee J, Kuang WJ, Rice GC, Wood WI. Structure and functional expression of a human interleukin-8 receptor (Reprinted from Science, Vol 253, pg 1278–1280, 1991). J Immunol 2009; 183: 2895–2897. 19696428

[bib36] Fu ZP, Chen X, Guan SW, Yan YJ, Lin H, Hua ZC. Curcumin inhibits angiogenesis and improves defective hematopoiesis induced by tumor-derived VEGF in tumor model through modulating VEGF-VEGFR2 signaling pathway. Oncotarget 2015; 6: 19469–19482. 26254223 10.18632/oncotarget.3625 PMC4637299

[bib37] Gu JW, Makey KL, Tucker KB, Chinchar E, Mao X, Pei I et al. EGCG, a major green tea catechin suppresses breast tumor angiogenesis and growth via inhibiting the activation of HIF-1alpha and NFkappaB, and VEGF expression. Vasc Cell 2013; 5: 9. 23638734 10.1186/2045-824X-5-9 PMC3649947

[bib38] Saha A, Kuzuhara T, Echigo N, Suganuma M, Fujiki H. New role of (−)-epicatechin in enhancing the induction of growth inhibition and apoptosis in human lung cancer cells by curcumin. Cancer Prev Res 2010; 3: 953–962. 10.1158/1940-6207.CAPR-09-0247 20606042

[bib39] Suganuma M, Saha A, Fujiki H. New cancer treatment strategy using combination of green tea catechins and anticancer drugs. Cancer Sci 2011; 102: 317–323. 21199169 10.1111/j.1349-7006.2010.01805.x

[bib40] Zhang Y, Yao K, Shi C, Jiang Y, Liu K, Zhao S et al. 244-MPT overcomes gefitinib resistance in non-small cell lung cancer cells. Oncotarget 2015; 6: 44274–44288. 26517520 10.18632/oncotarget.6236 PMC4792556

[bib41] Lu J, Zhao JM, Liu KD, Zhao J, Yang HY, Huang YT et al. MAPK/ERK1/2 signaling mediates endothelial-like differentiation of immature DCs in the microenvironment of esophageal squamous cell carcinoma. Cell Mol Life Sci 2010; 67: 2091–2106. 20221785 10.1007/s00018-010-0316-8 PMC11115913

[bib42] Jin GG, Yang Y, Liu HF, Liu KD, Zhao JM, Chen XH et al. Genome-wide analysis of the effect of esophageal squamous cell carcinoma on human umbilical vein endothelial cells. Oncol Rep 2016; 36: 155–164. 27222202 10.3892/or.2016.4816

[bib43] Lu J, Tang Y, Cheng Y, Zhang G, Yip A, Martinka M et al. ING4 regulates JWA in angiogenesis and their prognostic value in melanoma patients. Br J Cancer 2013; 109: 2842–2852. 24157826 10.1038/bjc.2013.670 PMC3844917

[bib44] Jin G, Zhao J, Yang YI, Liu K, Jiang Y, Zhang X et al. JAK/STAT3 signaling pathway mediates endothelial-like differentiation of immature dendritic cells. Oncol Lett 2015; 10: 3471–3477. 26788152 10.3892/ol.2015.3752 PMC4665379

[bib45] Lu J, Tang Y, Farshidpour M, Cheng YB, Zhang GH, Jafarnejad SM et al. JWA inhibits melanoma angiogenesis by suppressing ILK signaling and is an independent prognostic biomarker for melanoma. Carcinogenesis 2013; 34: 2778–2788. 24064223 10.1093/carcin/bgt318

[bib46] Jiang YA, Wu Q, Yang XW, Zhao JM, Jin YX, Li K et al. A method for establishing a patient-derived xenograft model to explore new therapeutic strategies for esophageal squamous cell carcinoma. Oncol Rep 2016; 35: 785–792. 26718633 10.3892/or.2015.4459

