# Supplementary material for: Combination curcumin and (−)-epigallocatechin-3-gallate inhibits colorectal carcinoma microenvironment-induced angiogenesis by JAK/STAT3/IL-8 pathway
Source: Oncogenesis. 2017 Oct 2;6(10):e384–. doi: 10.1038/oncsis.2017.84 (PMC5668882; doi:10.1038/oncsis.2017.84)
Supplement: Supplementary Figure Legends [file oncsis201784x1.doc]

**Supplementary figure legends**

**Figure S1. Colorectal carcinoma tissue homogenate CM enhanced the migration, invasion, tube formation and Dil-Ac-LDL uptake abilities of NECs.**  **(A-D)** NECs were induced by human colorectal carcinoma or peri-carcinoma tissue homogenate CM for 48 h. The migration, invasion, tube formation and Dil-Ac-LDL uptake abilities of NECs were examined by wound-healing assay (scale bar 40 μm) (A), transwell assay (scale bar 20 μm) (B), tube formation assay (scale bar 40 μm) (C) and Dil-Ac-LDL uptake assay (scale bar 20 μm) (D). Representative images were shown. Data are expressed as mean±SD from three independent experiments. ** p < 0.01, *** p < 0.001

**Figure S2. Colorectal carcinoma tissue homogenate CM promoted the transition of NECs toward TECs. (A-C)** NECs were induced by colorectal carcinoma or peri-carcinoma tissue homogenate CM for 48 h. The expression levels of TECs markers (TEM1, TEM8 and VEGFR2) were detected by qRT-PCR (A), Western blot (B) and immunofluoresence (scale bar 50 μm) (C). Results are expressed as mean±SD from three independent experiments. * p < 0.05, ** p < 0.01, *** p < 0.001.

**Figure S3. JAK/STAT3 signaling pathway was activated during the transition of NECs toward TECs induced by colorectal carcinoma tissue homogenate CM.** **(A)** NECs were induced by colorectal carcinoma or peri-carcinoma tissue homogenate CM for 48 h. The JAK and STAT3 levels were detected by qRT-PCR. **(B-C)** The phosphorylation levels of JAK and STAT3 were detected by Western blot (B) and immunofluoresence (scale bar 50 μm) (C). Results are expressed as mean±SD from three independent experiments. ** p < 0.01, *** p < 0.001.

**Figure S4. Combination curcumin and EGCG inhibited HT-29 CM-induced activation of JAK/STAT3/IL-8 signaling pathway.** The protein levels of p-JAK, p-STAT3 and IL-8 were detected by immunofluoresence (scale bar 50 μm). Data are shown as mean±SD from three independent experiments. * p < 0.05, ** p < 0.01, *** p < 0.001.

**Figure S5. Combination curcumin and EGCG inhibited HT-29 CM-induced transition of NECs toward TECs.** The protein levels of TECs markers (TEM1, TEM8, VEGFR2) were detected by immunofluoresence (scale bar 50 μm) from three independent experiments. Data are shown as mean±SD. *** p < 0.001.
