# Supplementary figures and images for: Combination curcumin and (−)-epigallocatechin-3-gallate inhibits colorectal carcinoma microenvironment-induced angiogenesis by JAK/STAT3/IL-8 pathway
Source: Oncogenesis. 2017 Oct 2;6(10):e384–. doi: 10.1038/oncsis.2017.84 (PMC5668882; doi:10.1038/oncsis.2017.84)

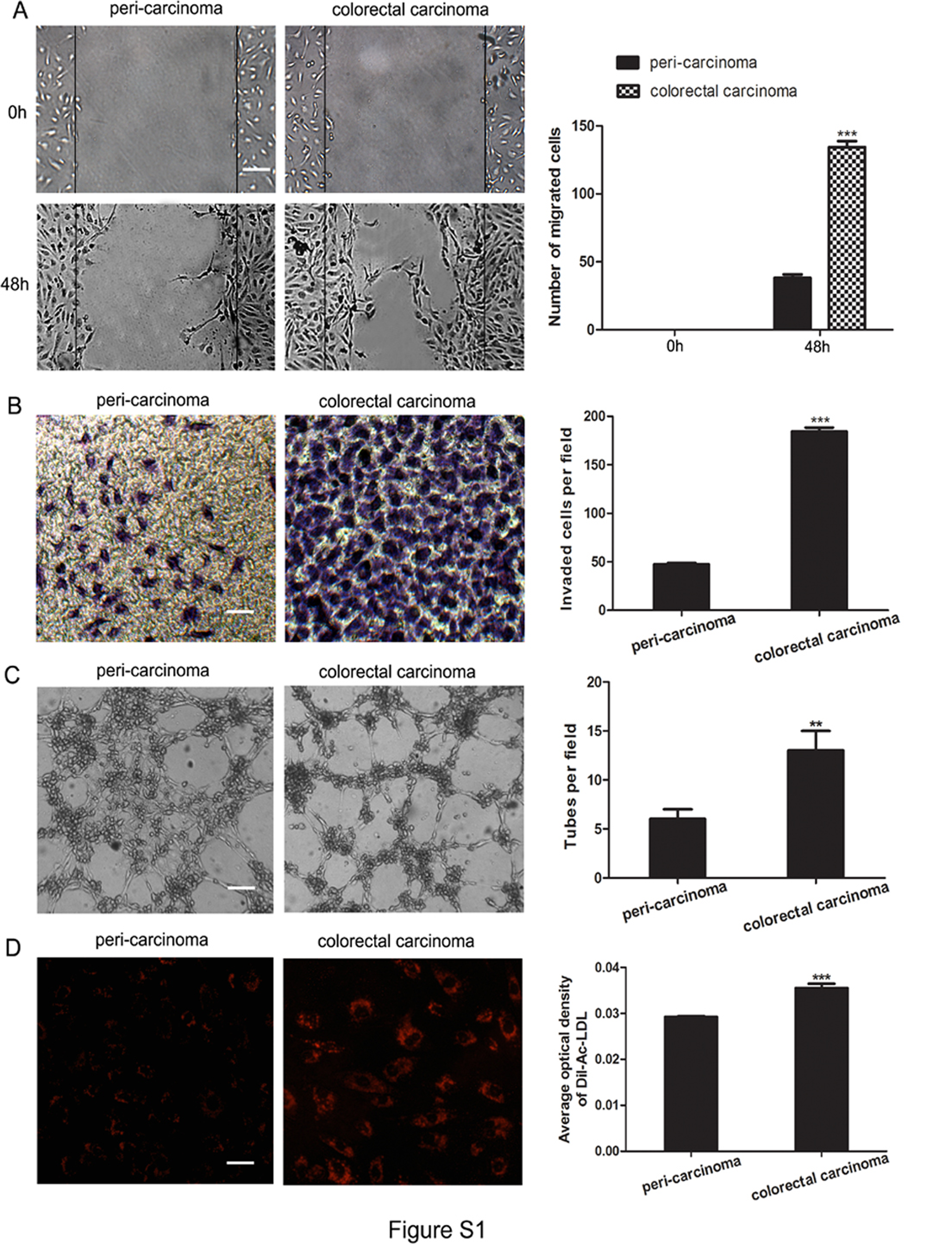

Supplement: Supplementary Figure 1 [file oncsis201784x2.tif]

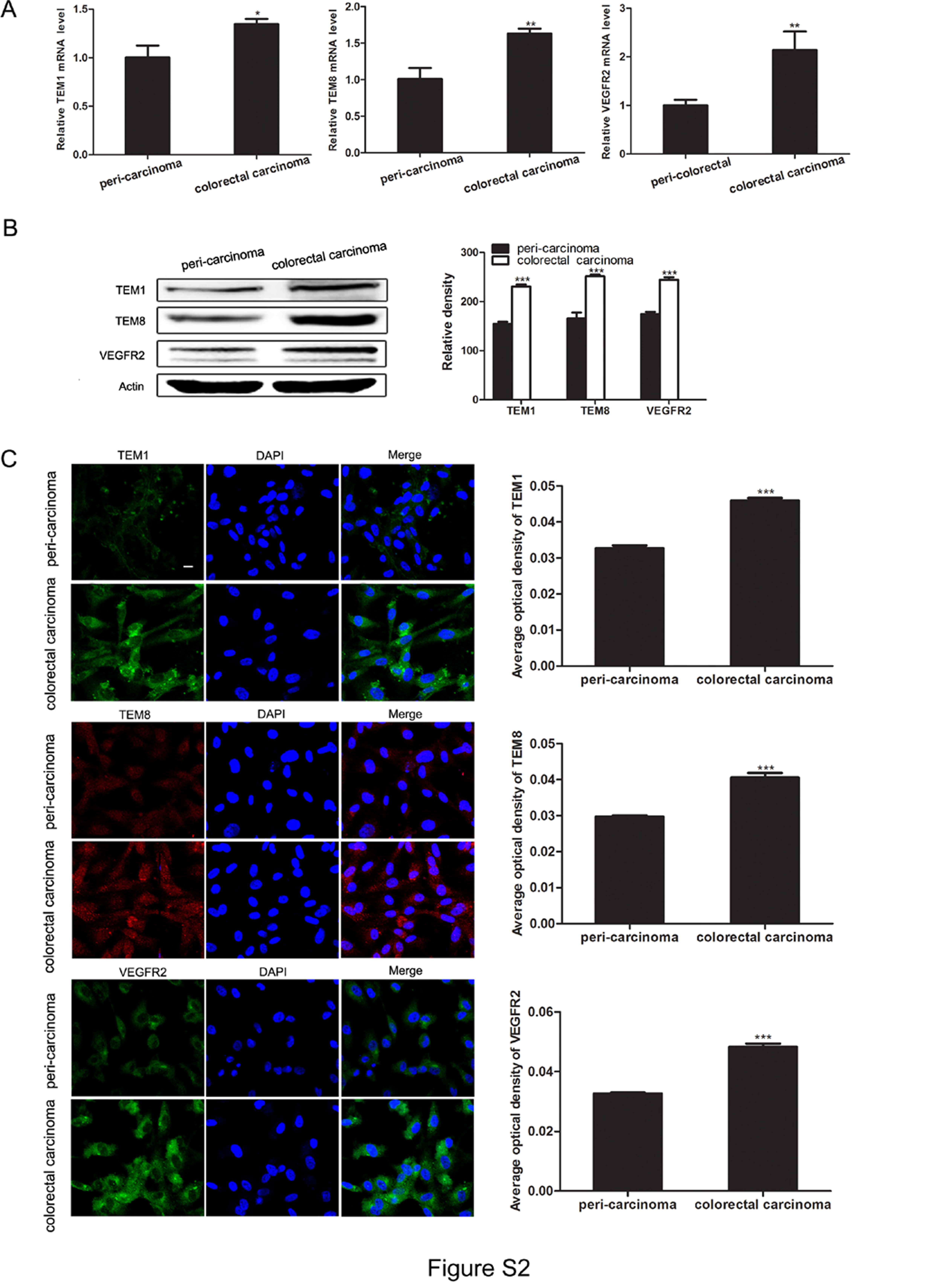

Supplement: Supplementary Figure 2 [file oncsis201784x3.tif]

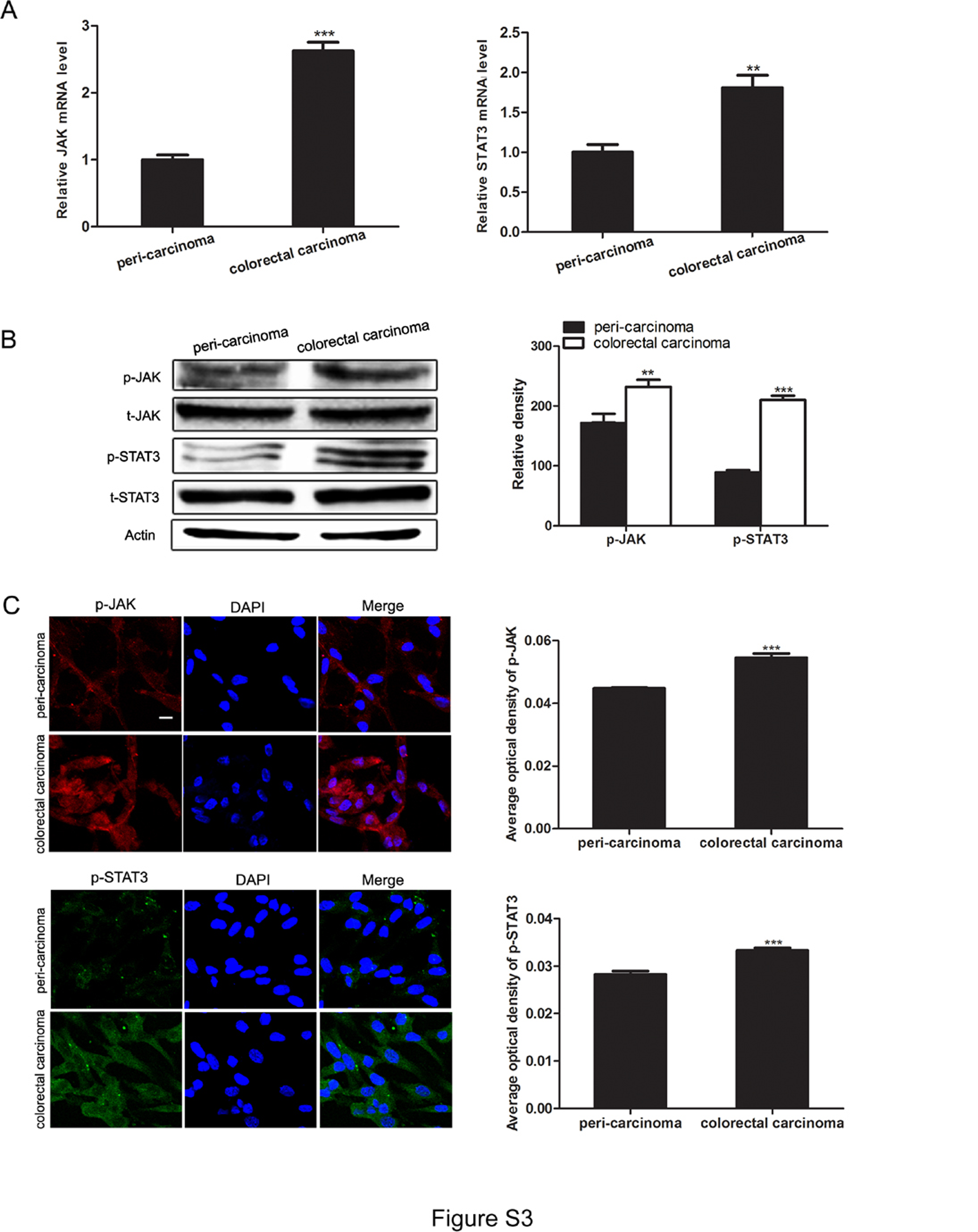

Supplement: Supplementary Figure 3 [file oncsis201784x4.tif]
